# MHCquant2 refines immunopeptidomics tumor antigen discovery

**DOI:** 10.1186/s13059-025-03763-8

**Published:** 2025-09-22

**Authors:** Jonas Scheid, Steffen Lemke, Naomi Hoenisch-Gravel, Anna Dengler, Timo Sachsenberg, Arthur Declerq, Ralf Gabriels, Jens Bauer, Marcel Wacker, Leon Bichmann, Lennart Martens, Marissa L. Dubbelaar, Sven Nahnsen, Juliane S. Walz

**Affiliations:** 1 Department of Peptide-based Immunotherapy, Institute of Immunology, University and University Hospital Tübingen, Tübingen, Germany; 2 Cluster of Excellence iFIT (EXC2180) “Image-Guided and Functionally Instructed Tumor Therapies”, University of Tübingen, Tübingen, Germany; 3 Quantitative Biology Center (QBiC), University of Tübingen, Tübingen, Germany; 4 Department of Computer Science, Biomedical Data Science, University of Tübingen, Tübingen, Germany; 5 Institute for Bioinformatics and Medical Informatics (IBMI), University of Tübingen, Tübingen, Germany; 6 Department of Computer Science, Applied Bioinformatics, University of Tübingen, Tübingen, Germany; 7 CompOmics, VIB Center for Medical Biotechnology, VIB, Ghent, Belgium; 8 Department of Biomolecular Medicine, Faculty of Medicine and Health Sciences, Ghent University, Ghent, Belgium; 9 German Cancer Consortium (DKTK) and German Cancer Research Center (DKFZ), partner site Tübingen, Tübingen, Germany; 10 Center for Systems and Engineering Immunology (CSEI), School of Medicine, Yale University, New Haven, CT USA; 11 BioOrganic Mass Spectrometry Laboratory (LSMBO), IPHC UMR 7178, University of Strasbourg, CNRS, ProFI FR2048, Strasbourg, France; 12 M3 Research Center, University Hospital of Tübingen, Tübingen, Germany; 13 Clinical Collaboration Unit Translational Immunology, Department of Internal Medicine, University Hospital Tübingen, Tübingen, Germany

**Keywords:** Immunopeptidomics, Nextflow, Nf-core, Pipeline, Mass spectrometry, HLA, Immunotherapy, Open-source

## Abstract

Confident identification of human leukocyte antigen (HLA)-presented peptides is crucial for advancing cancer immunotherapy. We present MHCquant2, a scalable and modular Nextflow pipeline integrated into nf-core as a reproducible, portable, and standardized workflow for immunopeptidomics. This integration allows a community-driven, robust solution for high-throughput analyses across operating systems and cloud infrastructures. MHCquant2 integrates open-source tools including OpenMS, DeepLC, and MS^2^PIP, improving peptide identifications by up to 27% across diverse MS platforms, particularly enriching low-abundant peptides. MHCquant2 demonstrates state-of-the-art performance on our novel benign_MHCquant2_ dataset (*n* = 92) and expands the benign human immunopeptidome by over 160,000 unique naturally presented HLA peptides.

**Supplementary Information:**

The online version contains supplementary material available at 10.1186/s13059-025-03763-8.

## Background

T cell recognition of human leukocyte antigen (HLA)-presented peptides plays a central role in the immune surveillance of malignant disease. Numerous immunotherapeutic approaches comprising cancer vaccines [1], adoptive transfer of T cells [2], and various T cell–targeting molecules aim to utilize respective tumor antigens to therapeutically induce an anti-tumor immune response [3–5]. Mass spectrometry (MS)-based immunopeptidomics provides insights into the antigenic landscape of malignant cells that go beyond sequencing- or binding affinity-based in silico predictions and thus facilitates the exploration of novel targets from a variety of antigens naturally processed and presented in cancer [6, 7]. These antigens comprise mutation-derived neoantigens [8, 9] as well as tumor-associated [10, 11] (TAA) and tumor-specific antigens from non-mutated protein products [12–14]. However, compared to conventional proteomics using tryptic digests, the low abundance and high variability of HLA-presented peptides pose distinct challenges to immunopeptidome analysis, with regard to sensitivity of detection and processing time [15, 16]. Technical and methodical advances, such as trapped ion mobility separation (TIMS), coupled with a time-of-flight mass analyzer (timsTOF) have significantly improved sensitivity and separation resolution in recent years [17–19]. Yet, these innovations substantially increase computational capacities, which hampers fast-track data processing.

Immunopeptidomics data processing pipelines typically involve database search, machine-learning-based re-evaluation, and false-discovery control, along with alignment and quantification to assess peptide abundance. The implementation of peptide property predictors such as DeepLC [20], MS^2^PIP [21], and Prosit [22, 23] has further enhanced immunopeptidomics sensitivity, leading to increased identification rates. However, this has also resulted in increased computing resource demands and extended runtime, particularly for large-scale datasets, calling for optimized pipelines to handle the complexity and scale of immunopeptidomics data. MHCquant1 [24] was written in Nextflow DSL1 [25] and improved the scalability and quantification of HLA-presented peptides for large datasets, but still falls short in sensitivity, hampering the detection of low-abundant antigens for immunotherapy development. FragPipe, with the MSFragger [26] search engine, introduced a fragment ion index algorithm that accelerates database searches and uses MSBooster [27] to enhance identifications, however, it is not simply deployable on high-performance computing infrastructures or cloud infrastructures. The closed-source PEAKS [28] pipeline leverages de novo sequencing-based features and deep learning for higher sensitivity but faces limitations in speed and resource efficiency, hindering large-scale tumor antigen identification. To cover the whole spectrum of personalized high-sensitive peptide detection in tumor samples to meta-analyses on large-scale immunopeptidomics datasets, a fast, sensitive, and standardized pipeline is essential. Here, we present MHCquant2, an open-source pipeline that implements OpenMS tools [29] and peptide property predictors (DeepLC, MS^2^PIP) for scalable and highly sensitive HLA peptide identification and quantification across various MS platforms. MHCquant2 is written in Nextflow DSL2 and developed as part of the nf-core initiative [30] for best-practice pipeline development. The integration into nf-core ensures not only reproducibility and portability but also long-term maintainability and transparent versioning, facilitating widespread adoption across operating systems, high-performance computing, and cloud environments.

First applications of MHCquant2 enabled (i) building a comprehensive benign reference repository, thereby allowing the refinement of non-mutated tumor-associated antigen definition, and (ii) the discovery of not yet described tumor antigens, comprising frequently presented self-antigens as well as low-abundant mutation-derived neoepitopes as potential targets for cancer immunotherapy.

## Results

### Peptide property predictors boost the identification and quantification of low-abundant HLA ligands in MHCquant2

**Fig. 1:**
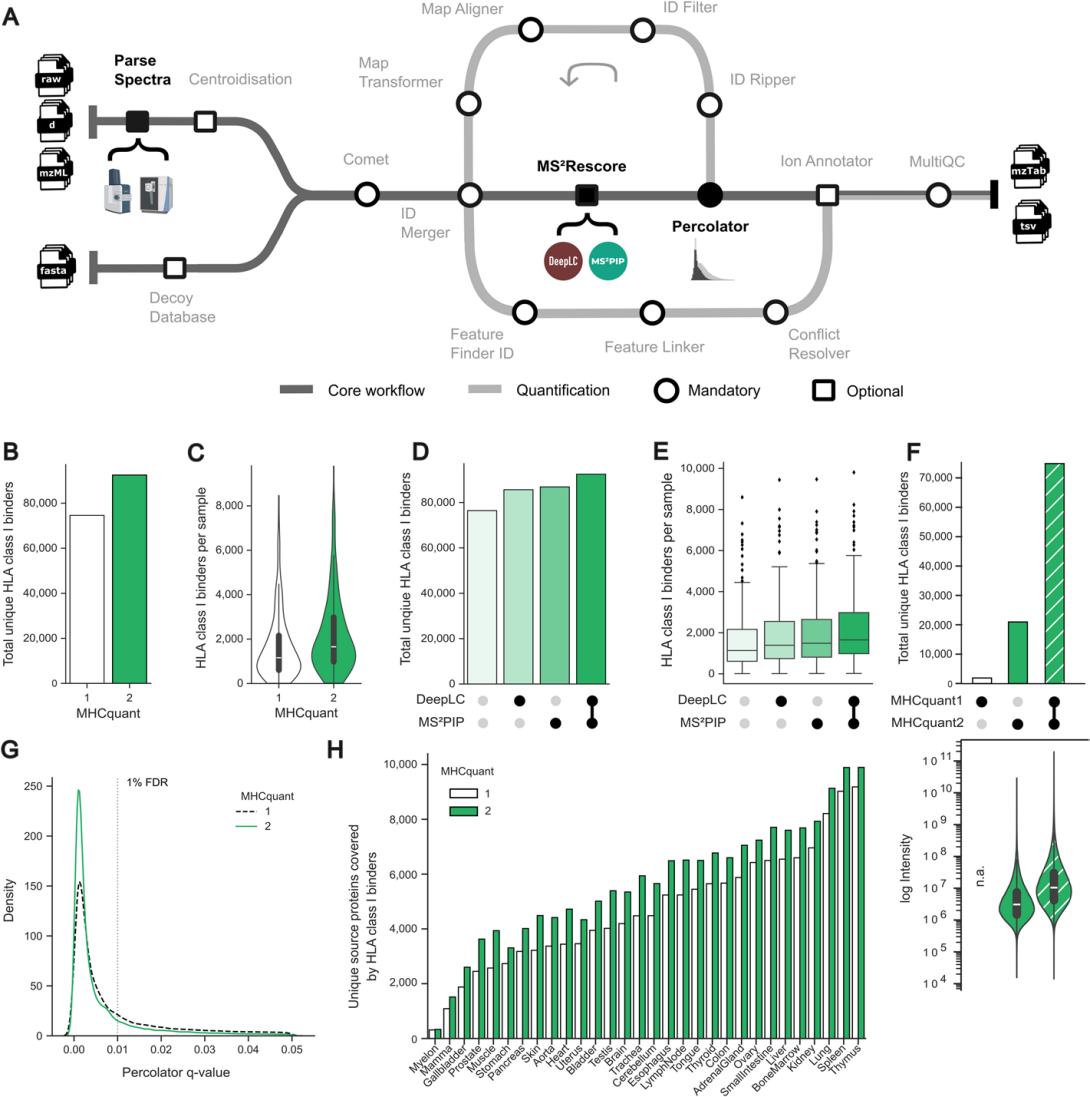
MHCquant2 workflow and HLA class I benchmark.** A** Subway plot of MHCquant2 key components. Each stop indicates a mandatory (o) or optional (▫) module of the pipeline. The dark gray subway represents the core workflow and the light gray subway the path of the quantification workflow. Stops indicated in black were implemented or extensively reworked within MHCquant2. **B-H** Benchmark against MHCquant1 using the HLA Ligand Atlas.** B** Total number of unique HLA class I binders across all samples and (**C**) distribution per sample identified using MHCquant1 and MHCquant2. **D** Total number of unique HLA class I binders and (**E**) distribution per sample identified without and with the feature generators DeepLC [20], MS^2^PIP [21], and their combination. Boxplots indicate the first to third quartile. Whiskers are defined as 1.5*IQR from the first and third quartile. **F** UpSet plot of total unique HLA class I binders identified by MHCquant1 and MHCquant2 shown at the top and their respective peptide intensity distribution displayed as a violin plot below. The inner boxplot of the violin plot depicts the median, first to third quartile of the distribution. **G** Density plot of Percolator q-value used as the FDR metric for MHCquant1 and MHCquant2. **H** Unique source proteins of HLA class I binders per tissue. Abbreviations: n.a., not available; FDR, false-discovery rate; IQR, interquartile range; HLA, human leukocyte antigen

The new MHCquant2 workflow is tailored toward identifying and quantifying MS-derived immunopeptidome data and uses well-established tools such as the search engine Comet [31], Percolator [32] for rescoring and false-discovery estimation as well as the OpenMS FeatureFinder [33] for quantification (Fig. 1A). MHCquant2 can now efficiently handle MS data input from the open mzML format [34] and different vendor formats, such as timsTOF data. The MS^2^Rescore framework was integrated to allow simple and scalable usage of DeepLC and MS^2^PIP to predict the retention time of peptides and fragment peak intensities of MS^2^ spectra. MHCquant2 efficiently streamlines these compute-intensive tasks by parallelizing processes in Nextflow, allowing fast analyses of large-scale data on workstations, high-compute clusters, and in the cloud.

To benchmark the MHCquant2 pipeline, we re-analyzed the HLA Ligand Atlas [35], a comprehensive resource of benign immunopeptidomics data. Compared to the published results of MHCquant1 [35], MHCquant2 increased the total number of uniquely identified HLA class I binders and HLA class II peptides using a 1% false-discovery rate (FDR) by 26.9% and 13.0%, respectively (Fig. 1B and Additional file 1: Fig. S1A). The median HLA class I binder identifications per sample increased from 1159 (range 9 to 8462) to 1644 (range 17 to 9806), and 2051 (range 150 to 12,620) to 2419 (range 145 to 14,017) for HLA class II peptides (Fig. 1C and Additional file 1: Fig. S1B). The combination of MS^2^PIP and DeepLC was identified as the primary source of the identification boost in the whole dataset (Fig. 1D and Additional file 1: Fig. S1C) and per tissue sample (Fig. 1E and Additional file 1: Fig. S1D). The overlap of HLA class I binders identified by MHCquant1 and MHCquant2 was 74,780 (76.6%), with 1877 (1.9%) MHCquant1-exclusive and 20,950 (21.5%) MHCquant2-exclusive (Fig. 1F). For HLA class II, 157,398 peptides (83.8%) were shared, 4738 peptides (2.5%) were MHCquant1-exclusive, and 25,833 peptides (13.7%) were MHCquant2-exclusive (Additional file 1: Fig. S1E). Notably, the median intensity of MHCquant2-exclusive HLA class I and II binders was 3.4-fold and 2.1-fold lower than the median intensity of the shared HLA class I binders, indicating that MHCquant2 enables the identification of low-abundant peptides (Fig. 1F and Additional file 1: Fig. S1E). Using MHCquant2, we observed an increase in the number of proteins covered by at least one HLA class I binder or HLA class II peptide. The per-tissue protein identifications covered by HLA class I binders increased from 4480 to 5758 (28.5%) and 2273 to 2495 (9.8%) for proteins covered by HLA class II peptides (Fig. 1H and Additional file 1: Fig. S1G).

We further investigated the Percolator q-value distribution reported by MHCquant1 and MHCquant2, which is used as the metric to assess the FDR. The increase in identifications is evident in the distribution of q-values below the 1% threshold, indicating a sensitivity boost only within the high-confidence range (Fig. 1G and Additional file 1: Fig. S1F). Analyses of Percolator feature weights, which attribute each feature an importance weight according to the target-decoy competition, revealed the highest cumulative feature weight (*N* = 71) for MS^2^PIP followed by Comet (*N* = 12) and DeepLC (*N* = 6) features. Specifically, feature *m0* from Comet and *rt_diff_best* from DeepLC are highly discriminative for HLA class I binders (Additional file 1: Fig. S2A and B). Correlation analysis and hierarchical clustering of the per-run feature weights reported by Percolator showed no apparent clusters, indicating that each feature holds valuable information to separate the target-decoy distribution by allowing the model to modify feature weights on a run-by-run basis (Additional file 1: Fig. S2C and D). In addition, no differences regarding amino acid frequency, hydrophobicity, and HLA allotype preference were observed for HLA class I binders and HLA class II peptides identified by the MHCquant1 and MHCquant2 pipelines (Additional file 1: Fig. S1H-M).

### MHCquant2 outperforms FragPipe and PEAKS on a novel benign_MHCquant2_ dataset

**Fig. 2:**
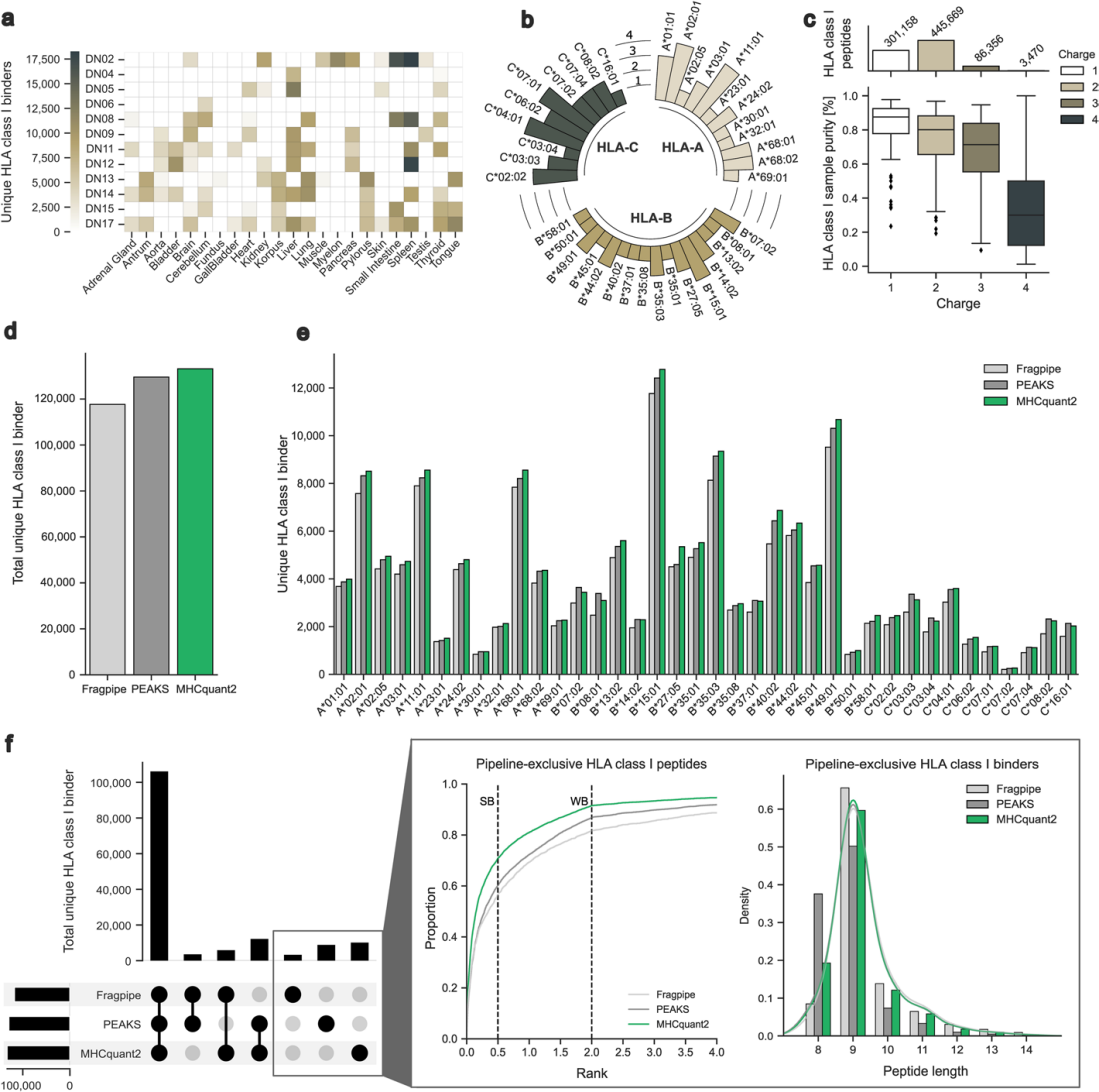
HLA class I immunopeptidome benchmark of MHCquant2, FragPipe, and PEAKS. The benchmark dataset (benign_MHCquant2_) was generated from various benign primary tissues. Metadata describing the cohort is documented in SDRF format (Additional file 2: Table S1)** A** Sample overview and HLA class I binder yield of benign_MHCquant2_ dataset. **B** HLA class I allotype distribution of all samples (*N* = 92). **C** Boxplot showing the distribution of HLA class I predicted binders and measured peptides ratio (purity) per charge state in the benign_MHCquant2_ dataset. The subplot above the boxplot indicates the number of HLA class I peptides per charge. **D** Total number and (**E***)* allotype annotated unique HLA class I binders identified using FragPipe, PEAKS, and MHCquant2 with the benign_MHCquant2_ dataset. **F** UpSet plot of identified HLA class I binders by FragPipe, PEAKS, and MHCquant2 (left). Cumulative density plot of NetMHCpan percentile ranks for pipeline-exclusive peptides with indicated SB and WB threshold (middle). Length distribution of pipeline-exclusive (bar) and total (line) HLA class I binders (right). Abbreviations: SDRF, Sample and Data Relationship Format; HLA, human leukocyte antigen; SB, strong binder; WB, weak binder

Benign immunopeptidome databases of human primary tissue are widely used as reference resources to identify HLA-presented antigen targets for cancer immunotherapy that show exclusive presentation on malignant cells to reduce the risk of autoimmune-related side effects [12, 18, 36]. Using the MHCquant2 pipeline, we generated a novel benign HLA class I and II immunopeptidome dataset from 92 human primary tissue samples (benign_MHCquant2_). Immunopeptidomics data was acquired on a timsTOF MS and processed using DeepLC and an improved timsTOF MS^2^PIP model [37] (Additional file 1: Fig. S4A). Using the improved timsTOF2024 MS^2^PIP model, we identified 133,163 HLA class I binders and 204,291 HLA class II peptides in the benign_MHCquant2_ dataset across 23 different primary tissues (Fig. 2A and Additional file 1: Fig. S3A). The median number of identifications was 6110 (range 366–18,188) for HLA class I and 6430 for HLA class II (range 1444–14,409). The HLA class I allotypes in the benign_MHCquant2_ dataset are distributed across 12 HLA-A, 16 HLA-B, and 10 HLA-C unique alleles and cover 99.6% of the world population with at least one allotype (Fig. 2B). The HLA class II allotypes are spread across 26 DR alleles and paired combinations of 12 DP and 23 DQ alleles covering 100% of the world population with at least one allotype (Additional file 1: Fig. S3C). HLA class I-presented peptides display an expected length distribution, with the majority being 9-mers (Additional file 1: Fig. S3D). The mass distribution ranges between 800 and 2000 Da across 4 charge states (range + 1 to + 4) with the majority accumulating around 1100 Da (Additional file 1: Fig. S3F). HLA class II peptides range between 800 and 3800 Da across 5 charge states (range + 1 to + 5, Additional file 1: Fig. S3G). The length distribution of HLA class II peptides displays two local maxima, occurring at approximately 9- and 15-mers. Singly-charged peptides mainly describe the first maximum, while the second maximum is composed of charges 2–5 (Additional file 1: Fig. S3E).

The identification of singly-charged ions is often challenging in immunopeptidomics due to the presence of chemical noise and undetectable uncharged fragments hindering accurate identification of these ions [6]. TIMS allows a better separation of peptides based on their charge state and collisional cross section, allowing the inclusion of singly-charged ions. Since singly-charged peptides represent a substantial proportion of HLA-presented peptides within the novel benign_MHCquant2_ dataset, we calculated the ratio of predicted binders and identified peptides (purity) per sample according to different charge states. The median ratio for singly-charged peptides was 87.6%, which is substantially higher than the ratios observed for charge + 2, + 3, and + 4 (80.1%, 71.4%, and 30.0%, Fig. 2C). 61,112 (30.5%) singly-charged HLA class I peptides were not detected with higher charge states indicating that the inclusion of singly-charged peptides represents a valuable source of HLA-presented peptides that were so far missed with standard immunopeptidomics methods (Additional file 1: Fig. S3H and I).

Using the benign_MHCquant2_ dataset, we benchmarked MHCquant2 against the two state-of-the-art pipelines FragPipe [27] and PEAKS [28]. A 13.2% and 2.8% increase in unique HLA class I binder identifications were reported by MHCquant2 (133,163 unique HLA class I binders) compared to FragPipe (117,676 unique HLA class I binders) and PEAKS (129,560 unique HLA class I binders), respectively. Unique HLA class II peptide identifications showed an increase of 5.1% of MHCquant2 (204,291 unique HLA class II peptides) compared to FragPipe (194,428 unique HLA class II peptides) and 1.6% compared to PEAKS (201,009 unique HLA class II peptides, Additional file 1: Fig. S4C). The increase in HLA class I binders is also reflected across HLA class I allotypes with a mean identification improvement of 13.8% compared to FragPipe and 2.0% compared to PEAKS (Fig. 2E). No pipeline-specific bias for HLA class I allotypes could be observed. Overlap analysis of identified peptides by each pipeline delineates 105,825 shared unique HLA class I binders and 171,698 shared HLA class II peptides (Fig. 2F and Additional file 1: Fig. S4D). 2992, 8552, and 9850 HLA class I binders were identified exclusively by FragPipe, PEAKS, and MHCquant2, respectively. Among these, MHCquant2 yielded 70.9% predicted strong binders (SB), 91.5% total binders (B) and 8.5% non-binders (NB), compared to FragPipe (56.9% SB, 81.7% B, 18.3% NB) and PEAKS (60.2% SB, 86.8% B, 13.2% NB). In contrast, 7740, 14,313, and 12,431 HLA class II peptides were found exclusively by the respective pipelines. MHCquant2-exclusive HLA class I binders report lower percentile ranks, indicating higher-affinity HLA-binding peptides compared to FragPipe- and PEAKS-exclusive peptides (Fig. 2F and Additional file 1: Fig. S4E). FragPipe- and MHCquant2-exclusive HLA class I binders show an expected length distribution that fits the overall length distribution. In contrast, PEAKS-exclusive 8-mer HLA class I binders tend to occur with a higher frequency. This preference for shorter peptides was also observed for the PEAKS-exclusive HLA class II peptides. Interestingly, FragPipe-exclusive HLA class II peptides tend to be longer than the overall length distribution (Additional file 1: Fig. S4D). The median GRAVY score was higher for PEAKS-exclusive HLA class I binders (median 0.43) compared to FragPipe (median 0.25) and MHCquant2 (median 0.06), indicating an increased hydrophobicity of these peptides (Additional file 1: Fig. S4F).

### MHCquant2 expands and refines tumor antigen discovery

**Fig. 3:**
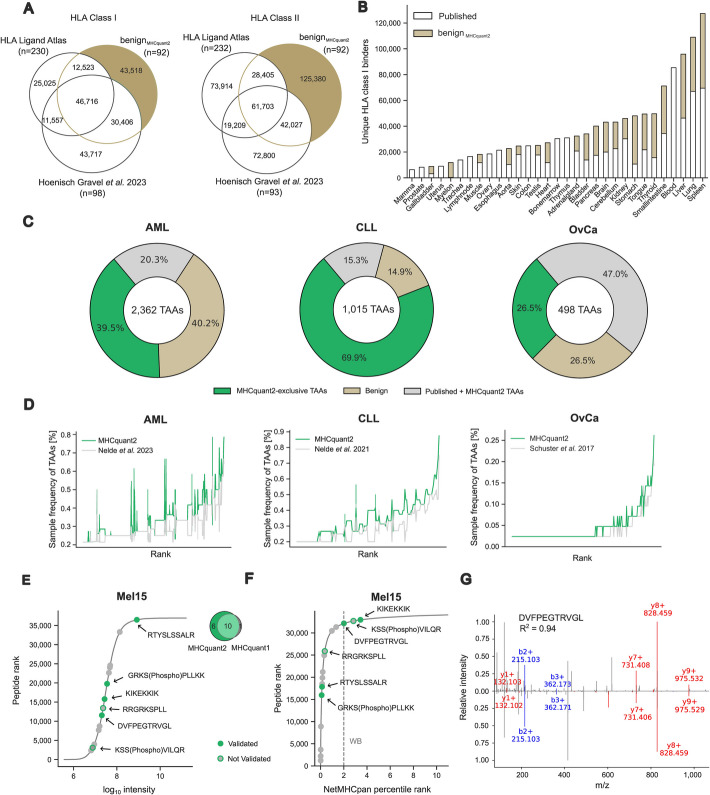
Refined tumor antigen discovery using MHCquant2. **A** Venn diagram depicting the HLA class I binder and HLA class II peptide overlap between the HLA Ligand Atlas [35], the Hoenisch Gravel et al. (PXD038782) dataset [18], and the benign_MHCquant2_ dataset. **B** Stacked bar plots showing the contribution of benign_MHCquant2_ HLA class I binders to public datasets according to primary tissue origin. **C** Comparison of published TAAs of AML [11], CLL [12], and OvCa [38] with re-analyzed TAAs by MHCquant2 and TAAs now identified in the new benign dataset. TAAs were defined according to the published filter criteria. **D** Sample frequency of shared HLA class I TAAs proposed by previous studies and identified by MHCquant2 for AML, CLL, and OvCa. TAAs are ranked according to sample frequency. **E** Intensity distribution of MHCquant2-identified peptides and neoepitopes of the melanoma dataset [8] compared to MHCquant1-identified neoepitopes in the respective dataset. MHCquant2-exclusive neoepitopes are annotated with their respective mutation location. **F** NetMHCpan percentile rank distribution of peptides and neoepitopes of the melanoma [8] dataset. **G** Mass-spectrometric neoantigen validation shown as mirror plot of experimentally eluted and synthetically validated spectrum of DVFPEGTRVGL (ENST00000353917 S296F, ENST00000360607 S337F, ENST00000372754 S419F, ENST00000372756 S378F) from one of the six detected neoepitopes in the melanoma dataset. Abbreviations: TAA, tumor-associated antigen; AML, acute myeloid leukemia; CLL, chronic lymphatic leukemia; OvCa, ovarian carcinoma; Mel, melanoma; HLA, human leukocyte antigen

To demonstrate the application potential of the MHCquant2 pipeline for antigen discovery in malignant disease, we combined our benign_MHCquant2_ dataset with previous benign immunopeptidome datasets [18, 35] re-analyzed by MHCquant2 into a comprehensive benign reference comprising 420 HLA class I samples with 213,462 unique HLA binders and 415 HLA class II samples with 423,438 HLA class II peptides (Fig. 3A). The benign_MHCquant2_ dataset contributed 43,518 new HLA class I binders and 125,380 new HLA class II peptides to the benign reference dataset. These novel identifications introduced or substantially extended the published benign reference datasets of various tissues such as myelon, stomach, and thyroid (Fig. 3B and Additional file 1: Fig. S5A). To further show the impact of the extended benign reference dataset in combination with the sensitive MHCquant2 pipeline in tumor antigen discovery, we re-analyzed published immunopeptidomic acute myeloid leukemia (AML) [11], chronic lymphatic leukemia (CLL) [12], and ovarian carcinoma (OvCa) [38] datasets and performed comparative analyses to exclude TAAs found in the benign reference and define novel TAAs identified by MHCquant2 (Fig. 3c and Additional file 1: Fig. S6B). 40.2% of published HLA class I AML TAAs were excluded by the extended benign reference, 20.3% were shared between public and MHCquant2-identified TAAs, and 39.5% of 2362 total TAAs were newly found by MHCquant2. HLA class I CLL TAAs were substantially extended to 1015 TAAs by 69.9% MHCquant2-exclusive TAAs and HLA class I OvCa TAAs to 498 by 26.5% MHCquant2-exclusive TAAs. Peptide frequency analysis of shared TAAs between published studies and the MHCquant2 re-analysis showed that HLA class I peptide frequencies are increased in 61.2% of CLL TAAs, 61.7% of AML TAAs, and 24.8% of OvCa TAAs (Fig. 3D and Additional file 1: Fig. S6C).

Beyond the identification of novel off-the-shelf tumor-exclusive antigens, we evaluated if the increased sensitivity of the MHCquant2 pipeline allows for the detection of low-abundant mutation-derived neoepitopes since we observed increased sensitivity of low-abundant peptides in benign tissues (Fig. 1F). We re-analyzed and quantified the mutation-informed melanoma dataset [8] with MHCquant2 and identified six additional neoepitopes compared to MHCquant1 (Fig. 3F) and generated Universal Spectrum Identifier (Additional file 2: Table S3). Interestingly, the majority of these neoepitopes accumulate in the lower abundant range (3/6 binding threshold < 2). Four of these neoepitopes could be validated by comparative measurement of synthetic peptides (Fig. 3G and Additional file 1: Fig. S6D).

Together, MHCquant2 provides a next-generation open-source immunopeptidome pipeline that enables parallel and highly sensitive processing of large-scale immunopeptidomics data for target antigen identification in cancer immunotherapy and beyond.

## Discussion

MS-based immunopeptidomics allows for the direct identification of naturally processed HLA-presented peptide antigens and thus provides invaluable information for immunotherapy design. Here, we present MHCquant2, an open-source nf-core pipeline that provides high-sensitive peptide identifications by integrating DeepLC and MS^2^PIP for accurate retention time and fragment intensity prediction across various MS platforms. The first application of the MHCquant2 pipeline expands benign references of HLA-presented peptides and facilitates the discovery of TAAs and neoepitopes.

Sensitivity enhancements in immunopeptidomics through peptide property predictors have been shown in previous studies [22, 23, 27, 39]. MHCquant2 combines the ability to accurately quantify HLA peptide abundances with peptide property predictors, enabling the detection of low-abundant HLA peptides, which is critical for identifying antigens that exhibit low expression levels [40]. By analyzing the HLA Ligand Atlas dataset [35], we observed a 26.9% increase and 13.0% increase in HLA class I binders and II peptides, respectively, over MHCquant1. We attributed these novel HLA peptides to low-abundant peptides that were previously indistinguishable, thereby lowering the in silico limits of detection and quantification. In line with other omics fields [41, 42], the addition of peptide property predictors, such as DeepLC and MS^2^PIP, increases sensitivity among high-confidence peptides below the 1% FDR threshold, indicating that these tools substantially contribute to identifying truly presented HLA peptides. Furthermore, feature weight analysis confirmed that each tool’s unique contribution enhances Percolator rescoring, aligning with the observed boost in identifications from each peptide property predictor. MHCquant2 achieves robust performance across various MS platforms, such as IMS-based MS, which was recently implemented for next-generation immunopeptidomics [18]. This enables the inclusion of high-quality singly-charged peptides provided by the additional IMS separation in timsTOF devices. Using a comprehensive timsTOF benchmark dataset, MHCquant2 outperformed the state-of-the-art pipelines FragPipe [27] and PEAKS [28]. Pipeline-exclusive analysis of HLA class I peptides further showed that MHCquant2 identifies more potent predicted binders, indicating higher quality identifications in line with previous reports on immunopeptidomics data quality protocols [43, 44]. FragPipe-exclusive peptides tend to be longer compared to the overall length distribution. This might be explained by FragPipe relying on features from MSBooster peptide property predictors [27], which might not be explicitly trained on timsTOF HLA class I and II data, potentially biasing towards tryptic length distributions [45]. In contrast, PEAKS-exclusive identifications showed a bias toward shorter peptide lengths, which may reflect methodological differences in peptide scoring. While the exact cause remains unknown, this could be influenced by in-source fragmentation events [46], false positives, or intrinsic challenges associated with the de novo-assisted database search strategy used by PEAKS.

For tumor antigen identification, the tumor-exclusive presentation without representation of the respective antigen on benign tissue is of crucial importance to avoid on-target-off-tumor adverse events and enable tumor-directed immune targeting. This prerequisite of tumor antigen discovery has led to the development of benign immunopeptidome repositories [18, 35, 47]. However, data processing of these repositories is not standardized, and the landscape of the whole benign immunopeptidome has yet to be fully explored. Using MHCquant2, we build a comprehensive benign tissue repository comprising re-analyzed data from available sources [18, 35] and the novel benign_MHCquant2_ dataset. This dataset builds on primary benign immunopeptidomes, enriching the benign repositories by more than 160,000 HLA class I- and HLA class II-presented peptides and expanding HLA allotypes and tissue sources. The first applications of this benign repository and the MHCquant2 pipeline enabled both, the refinement of TAAs identified in previous studies for multiple tumor entities [11, 12, 38] and the identification of novel, high-frequency non-mutated tumor-exclusive peptide antigens from these tumor entities. In addition to non-mutated tumor antigens, neoepitopes arising from tumor-specific mutations have been identified in recent years as the primary specificity of anti-cancer T cell responses induced by immune checkpoint inhibitors [48]. These neoepitopes were subsequently proposed as optimal candidates for T cell-based immunotherapy approaches [8, 16]. However, the low mutational burden of various tumor entities and the low abundance of peptides presented as HLA-restricted neoepitopes on tumor cells [49, 50] have hindered the MS-based identification and, consequently, the selection of neoepitopes for cancer immunotherapy. MHCquant2 allowed the identification of low-abundant mutation-derived HLA-presented peptides that were not discovered in previous studies [8, 24], suggesting that the increased sensitivity of this pipeline might further improve the detection of naturally presented neoepitopes. The subsequent prioritization of immunogenic neoepitopes can be further optimized using innovative proteogenomic approaches such as the NeoDisc pipeline [51].

As part of the modular nf-core framework [30] MHCquant2 allows for the simple integration of additional open-source tools such as the ion mobility predictor IM2Deep introduced in Tims^2^Rescore [37] or the de novo search engine Casanovo [52]. These tools could increase sensitivity even further and advance immunopeptidomics-guided antigen discovery. As of now, MHCquant2 has led to the development of the ‘Peptides for Cancer Immunotherapy Database’ (PCI-DB) [53], a comprehensive resource for cancer immunotherapy, which underlines the role of MHCquant2 as a best-practice immunopeptidomics pipeline.

## Conclusions

In this work, we present MHCquant2, a sensitive, scalable, and open-source pipeline for high-throughput identification and quantification of HLA-presented peptides. Developed within the nf-core framework, MHCquant2 ensures reproducibility, portability, and community-driven standardization, addressing a key unmet need for immunopeptidomics workflows. Applications of MHCquant2 and the benign_MHCquant2_ dataset enabled (i) refinement of tumor-associated antigens, (ii) discovery of novel, high-frequency tumor-exclusive peptides, and (iii) identification of low-abundant mutation-derived neoepitopes. Together, this best-practice pipeline advances sensitive large-scale immunopeptidome analysis and offers a robust open-source alternative to proprietary solutions. MHCquant2 is available on GitHub (https://github.com/nf-core/mhcquant/tree/2.6.0).

## Methods

### Sample collection

Benign solid tissue samples for the benign_MHCquant2_ dataset were collected within 72 h post-mortem during routine autopsies at the University Hospital Zürich. Subjects included in this study were not diagnosed with any malignant disease. The tissue was annotated by board-certified pathologists, snap-frozen in liquid nitrogen, and stored at − 80 °C.

### Isolation of HLA ligands

HLA class I and HLA class II molecules were isolated by previously described immunoaffinity chromatography protocols [44] using the pan HLA class I-specific W6/32 [54], pan HLA class II-specific Tü−39 [55], and HLA-DR-specific L243 [56] monoclonal antibodies. All antibodies were produced in-house at the Department of Immunology, University of Tübingen.

### timsTOF mass spectrometric data acquisition

Peptide separation was performed as previously described [18] on Bruker’s nanoElute LC system using an acclaim TM PepMap (Thermo Fisher Scientific, Waltham, USA) and a 75 μm × 25 cm Aurora Series emitter column (IonOpticks, Fitzroy, Australia). Sixty percent of the sample was injected in three technical replicates loading 5 μl. Peptides were separated along a gradient ranging from 0 to 95% Solvent B (AcN with 0.01% FA) over 60 min with consecutive ramps from 0 to 32% (30 min) and 32 to 40% (15 min), followed by two 5 min ramps to 60% and 95%, respectively. Eluting peptides were subsequently analyzed in the online-coupled trapped ion mobility spectrometry and time-of-flight mass spectrometer timsTOF Pro (Bruker Daltonics, Billerica, USA) equipped with a CaptiveSpray ion source using a data-dependent acquisition mode (DDA). PASEF ramps were set to 6, with an accumulation and ramp time of 200 ms. Mass range was set to 100–200 m/z with ion mobility ranging from 0.6 to 1.6 Vs/cm^2^; charge states above + 2 were included as well as + 1 > 600 m/z. The generated mass spectrometry raw data has been deposited in the ProteomeXchange Consortium database (https://www.proteomexchange.org) via the PRIDE partner repository [57] under dataset identifier PXD058436 (https://www.ebi.ac.uk/pride/archive/projects/PXD058436) [58]. The data was annotated in the Sample and Data Relationship Format (SDRF) with lesSDRF [59] to annotate ontologies and to ensure a simple re-usage of the provided data (Additional file 2: Table S1).

### Synthetic peptide validation

Non-phosphorylated synthetic peptides were produced using the standard 9-fluorenylmethyl-oxycarbonyl/tert-butyl strategy in a Liberty Blue Automated Peptide Synthesizer (CEM, Kamp-Lintfort, Germany). Peptides were cleaved from the resin using a TFA/triisopropylsilane/water (95%/2.5%/2.5% by vol.) mixture for 1 h, after which peptides were precipitated with diethyl ether and washed with diethyl ether thrice before resuspension in water ad lyophilization. Identity and purity were determined via C18-HPLC and LTQ Orbitrap XL MS (both Thermo Fisher Scientific). Phosphorylated synthetic peptides were produced by Intavis Peptide Services (Tübingen, Germany). Spectrum validation of the experimentally eluted peptides was performed by computing the similarity of the spectra with corresponding synthetic peptides measured in a complex matrix. A linear regression was fitted between all matching b and y ions of the MS/MS spectra of the eluted and the synthetic peptides to conduct the goodness of fit (*R*^2^). The generated mass spectrometry raw data has been deposited under dataset identifier PXD058436 (https://www.ebi.ac.uk/pride/archive/projects/PXD058436) [58].

### MHCquant2 processing workflow

MHCquant2 (v2.6.0) is implemented in Nextflow DSL2 and mainly comprises tools of the open-source software library OpenMS [29] (v3.1.0), which has been described previously [24]. Spectrum parsing from vendor formats to the open format mzML was done using tdf2mzml (v0.4) for timsTOF data and ThermoRawFileParser [60] (v1.4.3) for ThermoFisher devices. Identification and rescoring were performed using the OpenMS adapters to Comet [31] 2023.01 rev. 2 and Percolator [32] 3.5.0. The peptide property prediction framework MS^2^Rescore [39] (v3.0.1) was used to leverage DeepLC [20] (v2.2.27) and MS^2^PIP [37] (v4.0.0-dev8) including the new timsTOF2024 model. Feature alignment and quantification was conducted based on the post-Percolator FDR-filtered list using OpenMS’ chromatographic retention time aligner and FeatureFinder [33]. Finally, an ion annotation module was added, which allowed visualization and validation of neoepitopes by synthetic peptides. MHCquant is available via GitHub (https://github.com/nf-core/mhcquant/tree/2.6.0) [61] and Zenodo (https://zenodo.org/records/15194162) [62] under the MIT license. Comprehensive documentation of pipeline parameters, output, and usage is available via the nf-core website (https://nf-co.re/mhcquant/2.6.0).

### Comparison of MHCquant1 and MHCquant2

MHCquant2 (v2.6.0) was used to reprocess HLA class I and II data from the HLA Ligand Atlas [63]. The data was processed with the published search settings (Additional file 2: Table S2) and compared against the search results produced by MHCquant1 (v1.5.1) available on PRIDE (PXD019643). MHCquant2 additionally used the *feature_generators* flag to call DeepLC and MS^2^PIP via the MS^2^Rescore framework. MHCquant2 was then executed with four conditions: without DeepLC and MS^2^PIP, with DeepLC only, with MS^2^PIP only, and with both DeepLC and MS^2^PIP to evaluate the contributions of each feature generator to the identification rate. An additional quantification run was conducted with DeepLC and MS^2^PIP using the new *quantify* flag to investigate the intensity distribution of novel HLA class I and II peptides. To conduct the feature weight analysis, the absolute normalized feature weights of Percolator were used and sorted according to the respective feature source. The absolute normalized Percolator feature weights were correlated and clustered using hierarchical clustering. The GRAVY score was computed by the Biopython [64] package (v1.78). Peptides included in the comparison of allotype frequencies between MHCquant1 and MHCquant2 were annotated with the lowest predicted rank (< 2) of the samples’ respective HLA allotype.

### HLA binding prediction

Peptide binding predictions for HLA class I were conducted using the nf-core/epitopeprediction pipeline [65] (v2.3.1). NetMHCpan 4.1 [66] was specified as the prediction tool. HLA class I peptides were categorized as strong binders (percentile rank < 0.5), weak binders (percentile rank < 0.5 < x < 2) and non-binders (percentile rank ≥ 2).

### Evaluation and training of MS^2^PIP models

The dataset of Hoenisch Gravel et al. [18] (PXD038782) was downloaded from PRIDE. MHCquant version 2.5.0 was run with DeepLC to obtain the list of PSMs per sample and HLA class. The MS^2^PIP function *correlate* was used to predict MS^2^ peak intensities of PSMs and to correlate them with experimental peak intensities. The models timsTOF2023, Immuno-HCD, CIDch2, and TTOF5600 were used in this analysis.

Additionally, a new timsTOF model (timsTOF2024) was trained on the original training data [19] and supplemented with the HLA class II data (*N* = 376) of the PXD038782 dataset, as previously described [37]. Predicted and experimental MS^2^ peak intensities of the benign_MHCquant2_ dataset were computed and correlated, including the timsTOF2024 model.

### Benchmark of FragPipe, PEAKS, and MHCquant2 with benign_MHCquant2_ dataset

FragPipe (v21.1), PEAKS Studio (v11.5, Build 20,231,206), and MHCquant2 (v2.6.0) were used to conduct the benchmark analysis. The reference proteome from UniProtKB (Swiss-Prot, downloaded on 14.10.20) of *Homo sapiens* (TaxonID 9606) was in silico digested without enzymatic restriction. Database search was performed with a precursor mass tolerance of 20 ppm and a fragment mass tolerance of 0.02 Da. Methionine oxidation was specified as a variable modification, and a maximum of 3 and 5 modifications were allowed for HLA class I and II, respectively. The charge, mass range, and peptide length were set to 1–4, 800–2500 Da, and 8–14 for HLA class I and 1–5, 800–5000 Da, 12–30 for HLA class II. The seed in Percolator was fixed to 4711 to ensure reproducibility. A peptide-level FDR threshold of 1% among technical replicate sample groups was applied (Additional file 2: Table S2). For FragPipe, the Non-specific HLA workflow was adjusted to the same search settings, and MSBooster with Percolator was activated. Retention time and spectra prediction were turned on with the use of correlated features. Percolator was specified as the rescoring engine with default settings. For PEAKS, identical search settings were used in the Database Search workflow with deep learning-based features activated. The result data of FragPipe, PEAKS, and MHCquant2 has been deposited under dataset identifier PXD058436 (https://www.ebi.ac.uk/pride/archive/projects/PXD058436) [58].

### Description of benign_MHCquant2_ dataset

The benchmark results of MHCquant2 with the benign_MHCquant2_ dataset were used to describe the dataset in more depth. The IEDB population coverage tool [67] was used to obtain the World coverage for at least one HLA allotype in the dataset. The purity of a sample was calculated as the ratio between predicted HLA class I binder and total HLA class I peptides. Charge state overlap analysis on peptide- and PSM-level FDR was conducted using the Python package UpSetPlot (v0.9.0).

### Re-analysis of public immunopeptidomics benign and tumor-associated antigen studies

Re-analysis of published benign timsTOF data (PXD038782) [68] was carried out with the same configurations as in the benign_MHCquant2_ dataset processing protocol (Additional file 2: Table S2). Results from the HLA Ligand Atlas, PXD038782, and benign_MHCquant2_ were combined into a benign reference dataset. Immunopeptidomics studies of AML [11], CLL [12], OvCa [38], and Mel [8] were downloaded from PRIDE with the identifiers PXD038691 [69], PXD024871 [70], PXD007635 [71], PXD004894 [72] and re-analyzed with the exact data processing protocol as published (Additional file 2: Table S2). The downstream data analysis protocols of AML and CLL studies defined a TAA as a tumor-exclusive peptide with an allotype-specific sample frequency ≥ 20%. For the OvCa study, a TAA was defined according to a peptide originating from a source protein the authors postulated as TOP56 (HLA class I) and TOP32 (HLA class II) epithelial ovarian cancer-exclusive proteins. Peptides originating from these postulated proteins were compared against the re-analyzed peptides of these proteins by MHCquant2. Sticking to the previous data analysis protocol, TAAs identified by MHCquant2 and found in the benign reference dataset were excluded. TAAs from published studies now found in the benign reference dataset were highlighted. The reference database of PXD004894 was built using the published Ensemble identifiers and amino acid mutation. Protein sequences were retrieved from Ensemble release 78 using pyensemble [73] (v1.1.0). Mutated amino acids with 25 flanking amino acids of the respective proteins were obtained and added to the reference database.

## Supplementary Information

Additional file 1: Complementary figures of HLA class II data analyses; Percolator feature weight analysis for multiple datasets; Descriptive figures of benign_MHCquant2_ dataset; MS^2^PIP model performance analysis; Neoepitope spectra validation.

Additional file 2: SDRF annotations of the benign_MHCquant2_ dataset; MHCquant2 settings used for the analyzed datasets; USI of spectra matched to additional neoepitopes found by MHCquant2.

## Data Availability

The dataset generated during the current study is available in the PRIDE repository under the dataset identifier PXD058436 (https://www.ebi.ac.uk/pride/archive/projects/PXD058436), and metadata annotations in SDRF format are included in this published article (Additional file 2: Table S1). MHCquant2 is available on GitHub (https://github.com/nf-core/mhcquant/tree/2.6.0) and Zenodo (https:/zenodo.org/records/15194162) under the MIT license.
