# Supplementary material for: MHCquant2 refines immunopeptidomics tumor antigen discovery
Source: Genome Biol. 2025 Sep 22;26:290. doi: 10.1186/s13059-025-03763-8 (PMC12455830; doi:10.1186/s13059-025-03763-8)
Supplement: Supplementary file 1 — Additional file 1: Complementary figures of HLA class II data analyses; Percolator feature weight analysis for multiple datasets; Descriptive figures of benignMHCquant2 dataset; MS2PIP model performance analysis; Neoepitope spectra validation. [file 13059_2025_3763_MOESM1_ESM.docx]

**MHCquant2 refines immunopeptidomics tumor antigen discovery**

Jonas Scheid^1,2,3,4,5^, Steffen Lemke^1,2,3,4,5^, Naomi Hoenisch-Gravel^1,2^, Anna Dengler^1,2^, Timo Sachsenberg^5,6^, Arthur Declerq^7,8^, Ralf Gabriels^7,8^, Jens Bauer^1,2,9^, Marcel Wacker^1,2^, Leon Bichmann^10^, Lennart Martens^7,8,11^, Marissa L. Dubbelaar^1,2,3^†, Sven Nahnsen^2,3,4,5,12^†, Juliane S. Walz^1,2,9,13^†*

^1^Department of Peptide-based Immunotherapy, Institute of Immunology, University and University Hospital Tübingen, Tübingen, Germany.

^2^Cluster of Excellence iFIT (EXC2180) “Image-Guided and Functionally Instructed Tumor Therapies”, University of Tübingen, Tübingen, Germany.

^3^Quantitative Biology Center (QBiC), University of Tübingen, Tübingen, Germany.

^4^Department of Computer Science, Biomedical Data Science, University of Tübingen, Tübingen, Germany.

^5^Institute for Bioinformatics and Medical Informatics (IBMI), University of Tübingen, Tübingen, Germany.

^6^Department of Computer Science, Applied Bioinformatics, University of Tübingen, Germany.

^7^CompOmics, VIB Center for Medical Biotechnology, VIB, Ghent, Belgium.

^8^Department of Biomolecular Medicine, Faculty of Medicine and Health Sciences, Ghent University, Ghent, Belgium.

^9^German Cancer Consortium (DKTK) and German Cancer Research Center (DKFZ), partner site Tübingen, Germany.

^10^Center for Systems and Engineering Immunology (CSEI), School of Medicine, Yale University, New Haven, CT, USA.

^11^BioOrganic Mass Spectrometry Laboratory (LSMBO), IPHC UMR 7178, University of Strasbourg, CNRS, ProFI FR2048, Strasbourg, France

^12^M3 Research Center, University Hospital of Tübingen, Tübingen, Germany.

^13^Clinical Collaboration Unit Translational Immunology, Department of Internal Medicine, University Hospital Tübingen, Tübingen, Germany.

†These authors contributed equally to this work.

*Corresponding author: [juliane.walz@med.uni-tuebingen.de](mailto:juliane.walz@med.uni-tuebingen.de)

**Supplementary Figures**

**
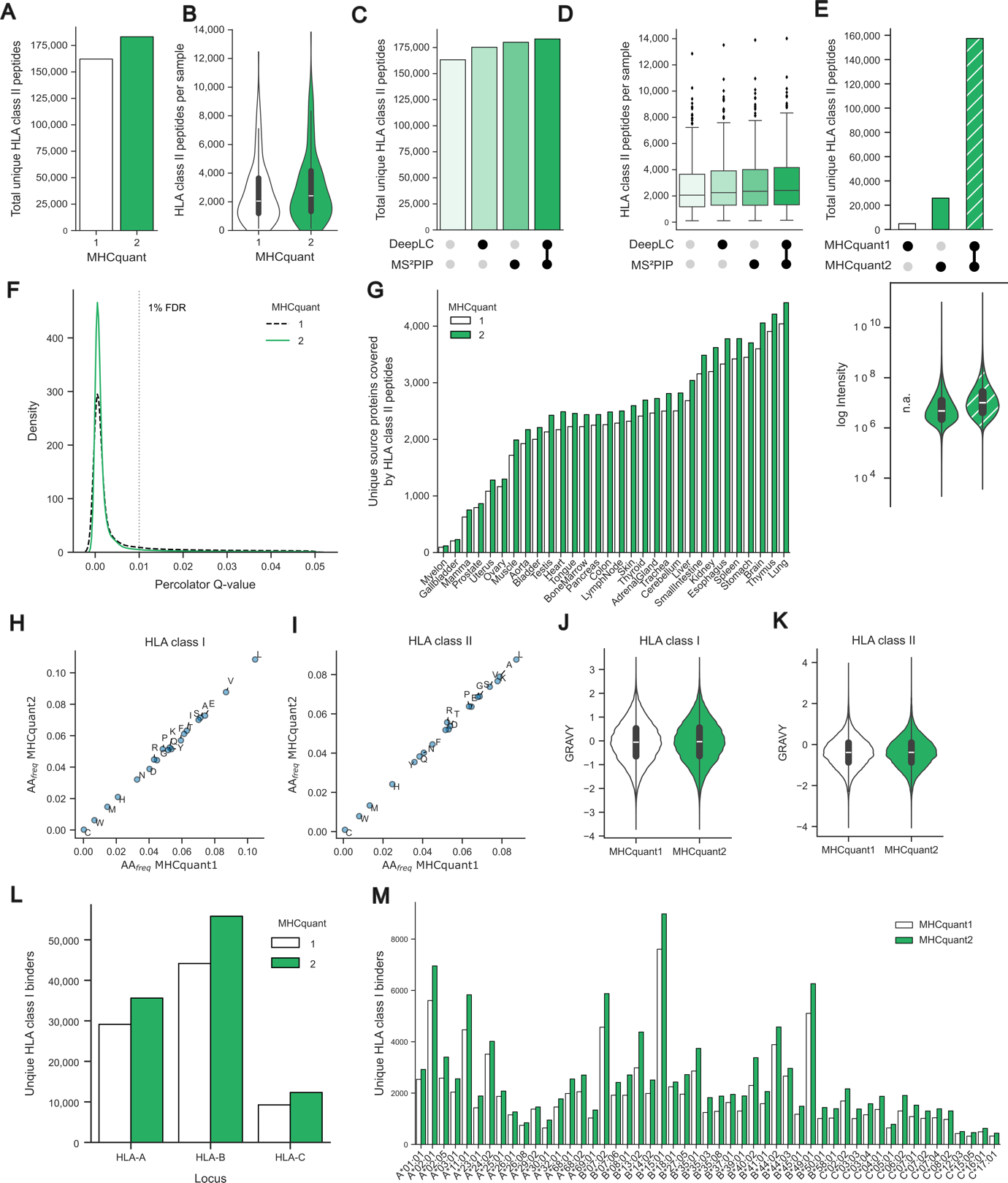
**

**Fig. S1 | HLA class II benchmark of MHCquant1 and MHCquant2.** The benchmark was conducted using the HLA Ligand Atlas. **A** Total number of unique HLA class II-presented peptides and **B** distribution per sample identified in MHCquant1 and MHCquant2. **C** Total number of unique HLA class II peptides across all samples and **D** distribution per sample identified with and without using the feature generators DeepLC, MS²PIP, and their combination. Boxplots indicate the first to third quartile. Whiskers are defined as 1.5*IQR (interquartile range) from the first to third quartile. **E** UpSet plot of total unique HLA class II-presented peptides identified by MHCquant1 and MHCquant2 shown at the top and their respective peptide intensity distribution displayed as a violin plot at the bottom. The inner box of the violin plot depicts the median, first, and third quartile of the distribution. **F** Density plot of Percolator q-value used as the FDR metric for HLA class II peptides with MHCquant1 and MHCquant2. **G** Unique source proteins of HLA class II-presented peptide per tissue. **H** Amino acid frequency of HLA class I binder and **I** HLA class II-presented peptides identified by MHCquant1 and MHCquant2. **J** Violinplot of GRAVY score distribution of HLA class I binders and **K** HLA class II-presented peptides. **L** Peptide-HLA class I annotation by the lowest rank of NetMHCpan 4.1 per locus and **M** allele. Abbreviations: GRAVY: grand average of hydropathy; HLA: Human leukocyte antigen.


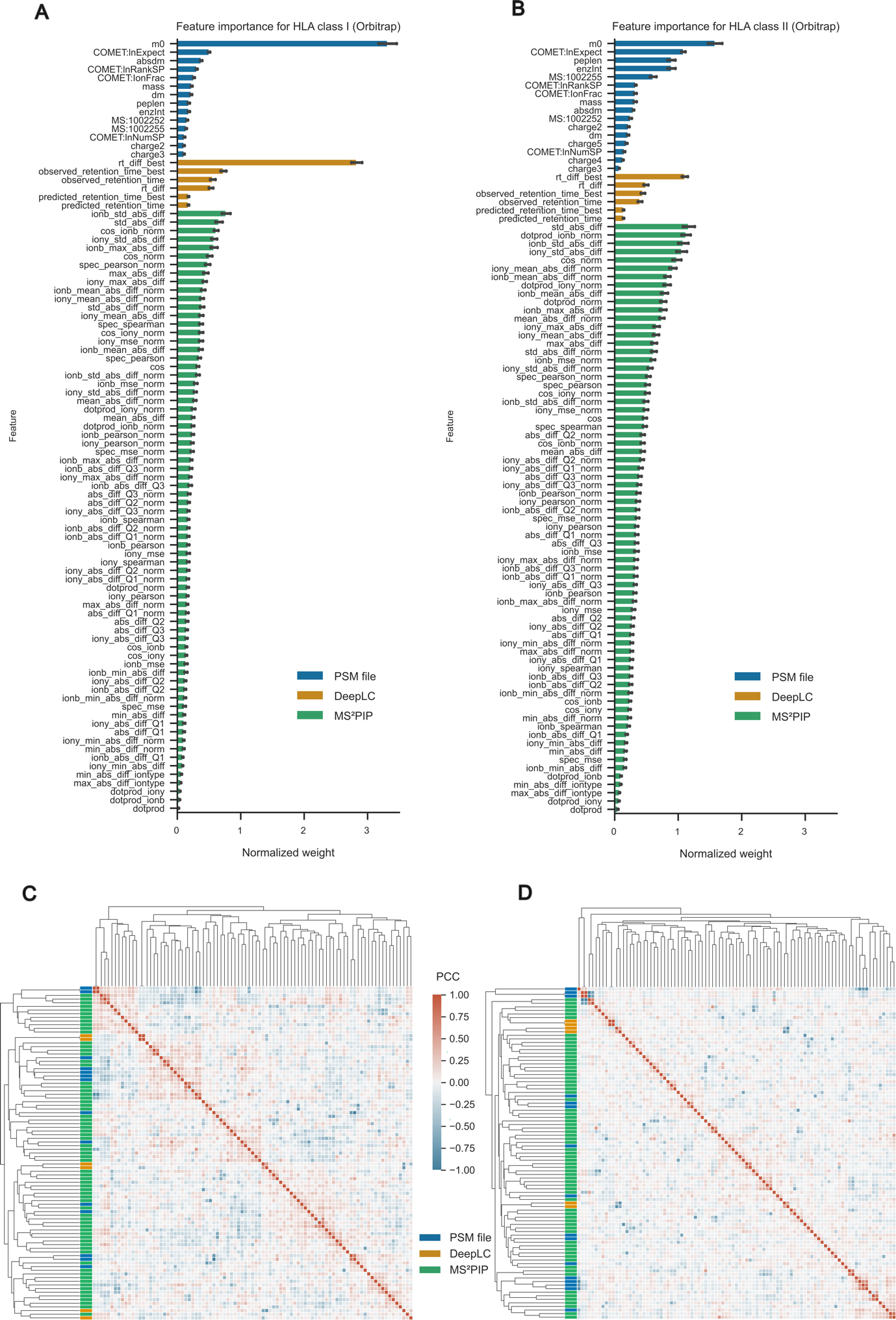


**Fig. S2 | Percolator feature weight analysis.** All data was generated using the HLA Ligand Atlas dataset. **A** Barplot of normalized feature weights per MS run according to the Comet (PSM file), DeepLC, and MS²PIP for HLA class I and **B** HLA class II. Each bar is presented with a 95% confidence interval error bar for the mean of each feature. **C** Hierarchical clustering of Pearson correlation coefficient (PCC) of normalized feature weights per MS run according to Comet (PSM file) DeepLC, and MS²PIP for HLA class I and **D** HLA class II. Abbreviations: PSM: Peptide-Spectrum match; HLA: Human leukocyte antigen.


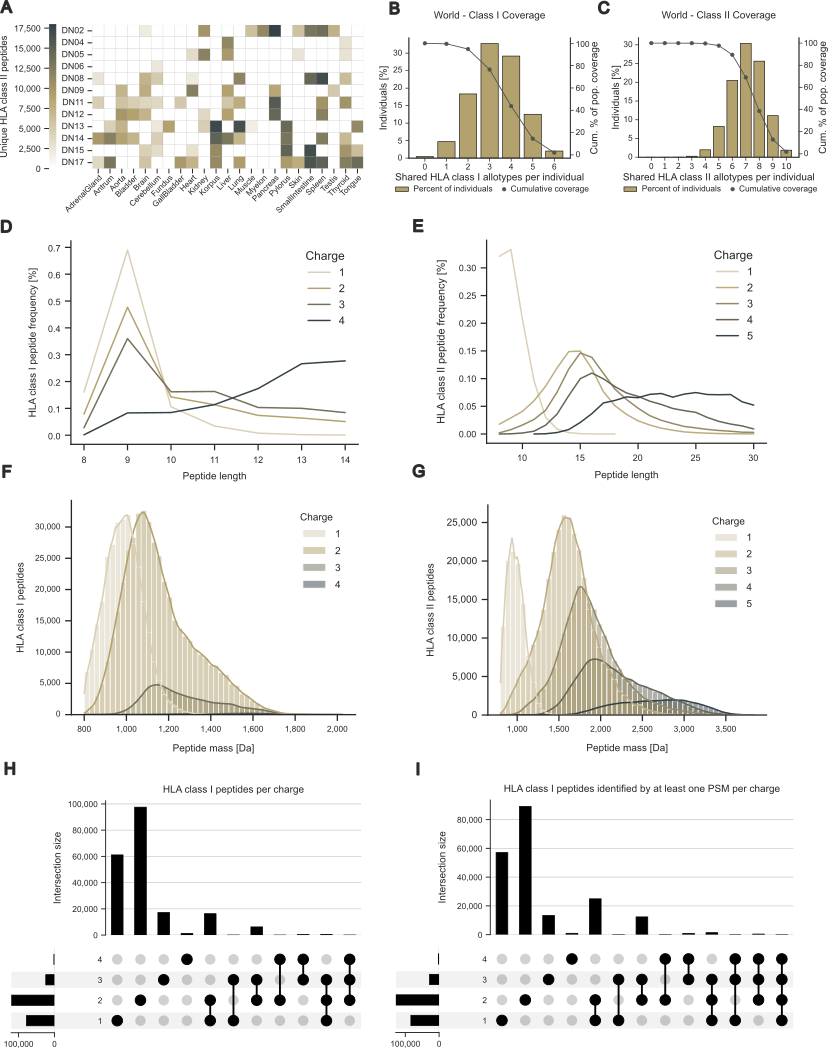


**Fig. S3 | Characterization of benign_MHCquant2_ dataset. A** Sample overview and HLA class II-presented peptide yield. **B** Population coverage of the HLA class I and **C** HLA class II allotypes of the benign_MHCquant2_ dataset cohort compared to the world population determined by the IEDB population coverage tool. Frequencies of individuals within the world population carrying up to 6 HLA class I and 10 HLA class II allotypes (x-axis) are indicated as bars on the left y-axis. The cumulative percentage of population coverage is depicted as dots on the right y-axis.

**D** Length distribution of HLA class I- and **E** HLA class II-presented peptides according to charge states. **F** Histogram of mass distribution in Dalton paired with a kernel density estimation of HLA class I- and **G** HLA class II-presented peptides. **H** UpSet plot of HLA class I peptide charge states using peptide-level FDR and **I** using PSM-level FDR, where at least one PSM per charge was identified. Abbreviations: IEDB: Immune Epitope Database; FDR: false-discovery rate; PSM: peptide-spectrum match; HLA: Human leukocyte antigen.


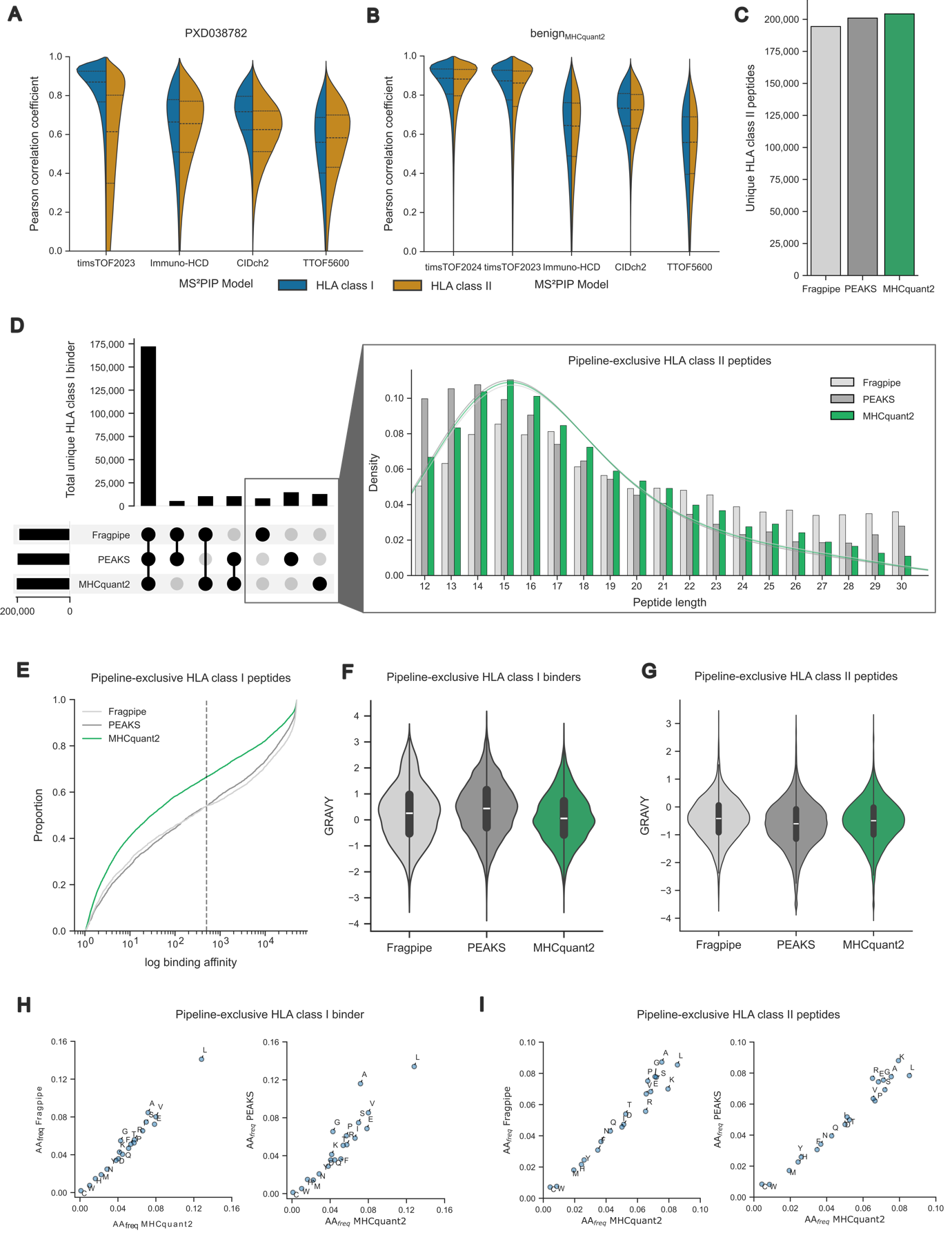


**Fig. S4 | MS^2^PIP model evaluation and HLA class II immunopeptidome benchmark of MHCquant2, FragPipe, and PEAKS. A** Pearson correlation of experimental and predicted peak intensities of the Hoenisch Gravel *et al.* dataset (PXD038782) and **B** of the benign_MHCquant2_ including the timsTOF20204 model. **C** Total number of unique HLA class II peptides of the benign_MHCquant2_ dataset identified by FragPipe, PEAKS, and MHCquant2. **D** UpSet plot of intersection size between identified HLA class II-presented peptides identified by FragPipe, PEAKS, and MHCquant2. Length distribution of pipeline-exclusive (bar) and total (line) HLA class II-presented peptides. **E** Cumulative density plot of NetMHCpan binding affinity for pipeline-exclusive HLA class I peptides. The IC50 threshold smaller or equal to 500 defining a binder is indicated by the vertical line. **F** Violinplot depicting the GRAVY score distribution of pipeline-exclusive HLA class I binders and **G** HLA class II-presented peptides. **H** Amino acid frequency of pipeline-exclusive HLA class I binders comparing MHCquant2 with FragPipe and PEAKS, respectively. **I** Amino acid frequency of pipeline-exclusive HLA class II-presented peptides comparing MHCquant2 with FragPipe and PEAKS, respectively. Abbreviations: GRAVY: grand average of hydropathy; IC50: Half-maximal inhibitory concentration; HLA: Human leukocyte antigen.


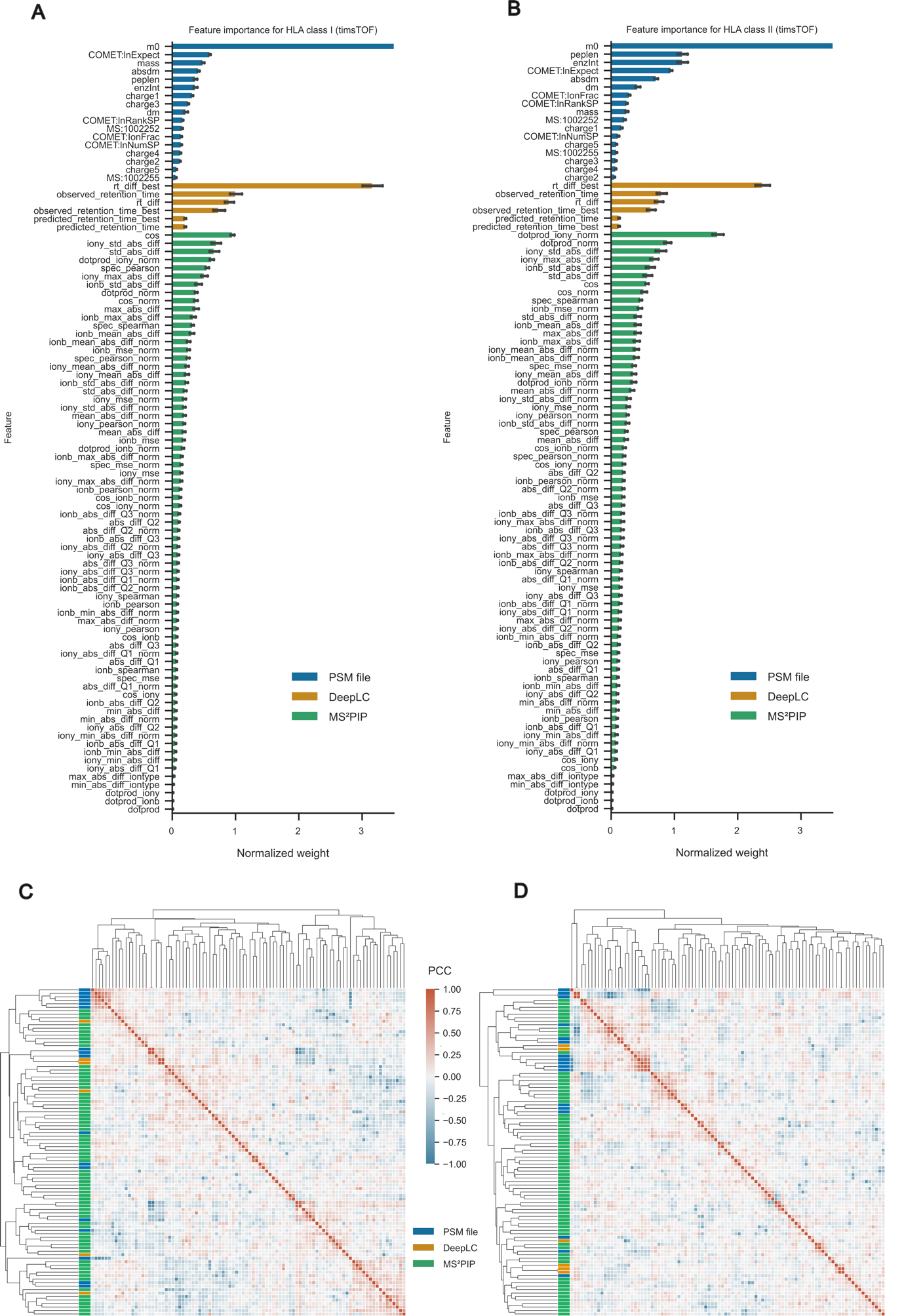


**Fig. S5 | Percolator feature weight analysis with the benign_MHCquant2_ dataset . A** Barplot of normalized feature weights per MS run according to the Comet (PSM file), DeepLC, and MS²PIP for HLA class I and **B** HLA class II**.** Each bar is presented with a 95% confidence interval error bar for the mean of each feature. **C** Hierarchical clustering of Pearson correlation coefficient (PCC) of normalized feature weights per MS run according to Comet (PSM file) DeepLC, and MS²PIP for HLA class I and **D** HLA class II. Abbreviations: PSM: Peptide-Spectrum match; HLA: Human leukocyte antigen.


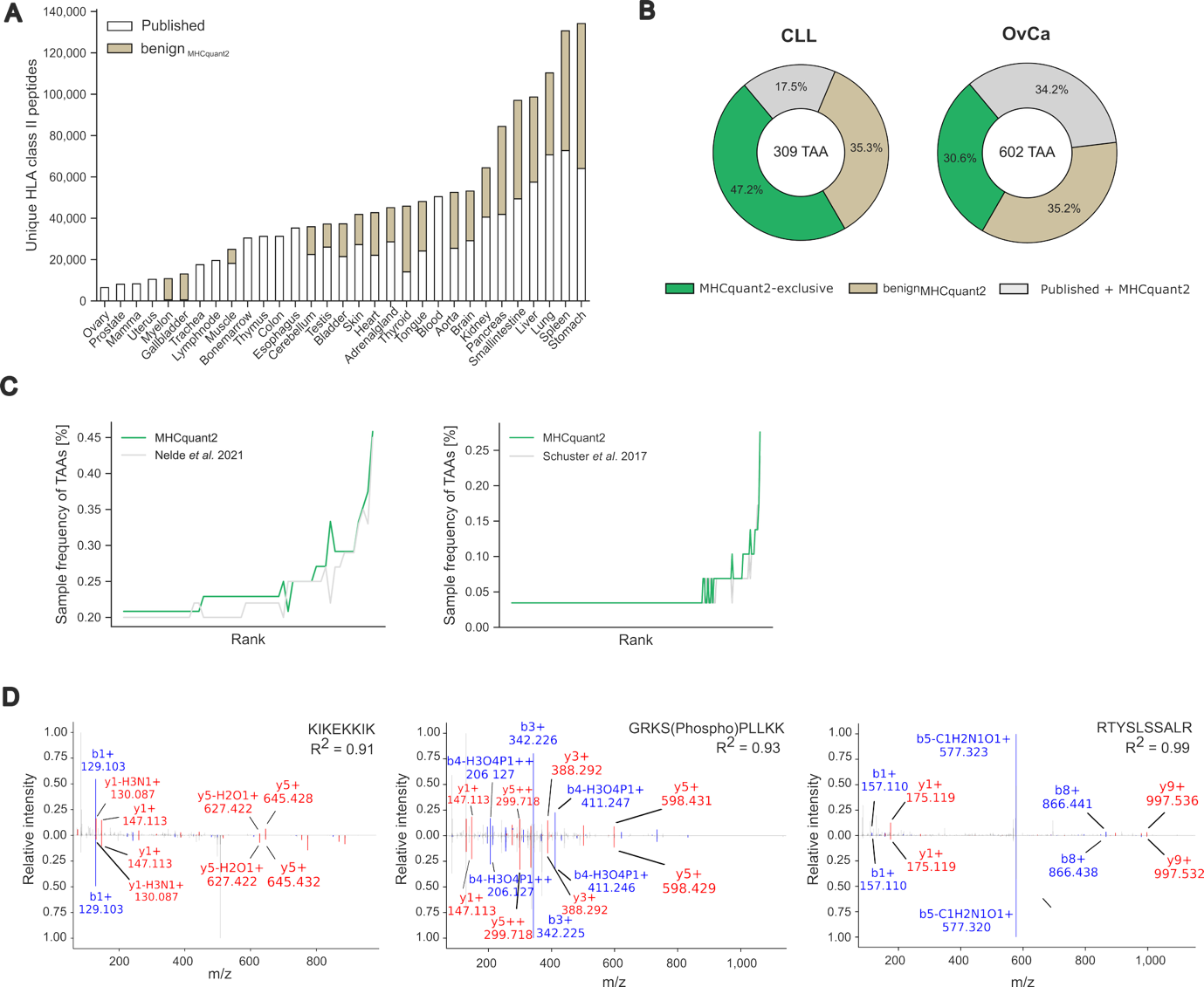


**Fig. S6 | Refined tumor-associated antigen discovery using MHCquant2.**

**A** Stacked bar plots showing the contribution of benign_MHCquant2_ HLA class II peptides to previous datasets according to primary tissue origin. **B** Comparison of published TAAs of CLL and OvCa with re-analyzed TAAs by MHCquant2 and TAAs now identified in new benign datasets. TAAs were defined according to the published filter criteria. **C** Sample frequency of shared HLA class II TAAs proposed by public studies and identified by MHCquant2 for CLL and OvCa. TAAs are ranked according to sample frequency. **D** Mass-spectrometric neoantigen validation shown as mirror plots of experimentally eluted and synthetically validated spectra of GRKS(Phospho)PLLKK (ENST00000369367 P383S, ENST00000465950 P68S, ENST00000549162 P191S), KIKEKKIK (ENST00000518476 E304K, ENST00000620844 E160K), RTYSLSSALR (ENST00000224237 G41S) of three of the six newly detected neoepitopes in the melanoma dataset by MHCquant2. Abbreviations: TAA: tumor-associated antigen CLL: chronic lymphatic leukemia, OvCa: ovarian carcinoma; HLA: Human leukocyte antigen.
